# Emergency Department Visits by Incarcerated Adults for Nonfatal Injuries — United States, 2010–2019

**DOI:** 10.15585/mmwr.mm7211a2

**Published:** 2023-03-17

**Authors:** Avital Wulz, Gabrielle Miller, Livia Navon, Jill Daugherty

**Affiliations:** 1 Division of Injury Prevention, National Center for Injury Prevention and Control, CDC.

During 2010–2019, U.S. correctional authorities held 1.4–1.6 million persons in state and federal prisons annually, and 10.3–12.9 million persons were admitted to local jails each year (*1*,*2*). Incarcerated persons experience a disproportionate burden of negative health outcomes, including unintentional and violence-related injuries (*3*,*4*). No national studies on injury-related emergency department (ED) visits by incarcerated persons have been conducted, but a previous study demonstrated a high rate of such visits among a Seattle, Washington jail population (*5*). To examine nonfatal injury-related ED visits among incarcerated adults, CDC analyzed 2010–2019 National Electronic Injury Surveillance System–All Injury Program (NEISS-AIP) data. During 2010–2019, an estimated 733,547 ED visits by incarcerated adults occurred in the United States. The proportion of ED visits resulting from assault* and self-harm among incarcerated adults was five times as high as those among nonincarcerated adults. Among incarcerated adults, men and adult persons aged <65 years had the highest proportion of assault-related ED visits. Falls accounted for the most ED visits among incarcerated adults aged ≥65 years. A higher proportion of ED visits by incarcerated women than incarcerated men were for overdose or poisoning. These findings suggest that injuries among incarcerated adults differ from those among nonincarcerated adults and might require development and implementation of age- and sex-specific prevention strategies for this population.

NEISS-AIP collects data on patients treated in EDs for nonfatal injuries from a nationally representative, stratified probability sample of hospitals.^†^ Data are weighted by the inverse probability of selection to provide annual national estimates. A visit by an incarcerated person was defined as an ED visit by a person aged ≥18 years who was transported to an ED from a jail or prison for an injury.^§^ A visit by a nonincarcerated person was defined as an ED visit by any other persons aged ≥18 years. Data include a narrative summarizing the circumstances of each visit written by a trained data abstractor. Specific terms within narratives were used to identify visits by incarcerated persons.^¶^ An iterative process was used to improve identification of visits by incarcerated persons through manual review of a sample of narratives by two authors to ensure that selected visits met the case definition and to identify additional terms.

The weighted number of ED visits among incarcerated and nonincarcerated adults were calculated using SAS-callable SUDAAN (version 11.0.1; RTI International). Visits were stratified by patient sex, age group, injury intent, mechanism of injury,** and disposition,^††^ and the proportion of visits with these characteristics was calculated separately for incarcerated and nonincarcerated adults. Ratio of proportions (RPs) with 95% CIs were calculated to compare ED visits by incarcerated and nonincarcerated adults. Rao-Scott chi-square tests were used to calculate p-values, and p-values <0.05 were considered statistically significant. SUDAAN Rlogist procedure was used to estimate RPs with 95% CIs by sex and age group among incarcerated adults. This activity was reviewed by CDC and was conducted consistent with applicable federal law and CDC policy.^§§^

During 2010–2019, an estimated 733,547 ED visits by incarcerated adults and 211,497,918 by nonincarcerated adults occurred in the United States (Table 1). Compared with ED visits among nonincarcerated adults, a higher proportion of ED visits among incarcerated adults were among men (83.7% versus 50.9%) and adults aged <45 years (77.1% versus 51.0%). The proportion of visits due to assault and self-harm was about five times as high among incarcerated adults (34.6% and 9.1%, respectively) than among nonincarcerated adults (6.5% and 1.9%, respectively). The most common mechanism of injury among incarcerated adults with an ED visit was being struck by or against an object (44.0%); among nonincarcerated adults, this mechanism accounted for 14.7% of ED visit injuries. The most common mechanisms of injury among nonincarcerated adults with an ED visit were “other” mechanisms (e.g., transportation-related injuries and drowning) (41.0%). A higher proportion of ED visits by incarcerated adults resulted in hospitalization or transfer to another hospital (17.3%) than did ED visits by nonincarcerated adults (13.2%).

**TABLE 1:** Estimated number of nonfatal injury-related emergency department visits among incarcerated and nonincarcerated adults — National Electronic Injury Surveillance System–All Injury Program, United States, 2010–2019

Characteristic	Incarcerated		Nonincarcerated		RP^†^	p-value
	No.*	% (95% CI)	No.	% (95% CI)		
**Total**	**733,547**	**—**	**211,497,918**	**—**	**—**	**—**
**Sex**						
Men	614,174	83.7 (78.9–88.5)	107,723,904	50.9 (48.9–53.0)	1.64	<0.001
Women	119,373	16.3 (11.5–21.1)	103,771,368	49.1 (47.0–51.1)	0.33	<0.001
**Age group, yrs**						
18–24	146,858	20.0 (17.4–22.7)	33,850,167	16.0 (15.0–17.0)	1.25	0.004
25–34	261,062	35.6 (33.7–37.5)	41,261,154	19.5 (18.6–20.4)	1.82	<0.001
35–44	158,055	21.5 (20.2–22.9)	32,846,662	15.5 (14.9–16.2)	1.39	<0.001
45–54	95,325	13.0 (11.7–14.3)	33,670,314	15.9 (15.1–16.7)	0.82	<0.001
55–64	46,951	6.4 (5.1–7.7)	26,762,203	12.7 (12.4–12.9)	0.51	<0.001
≥65	25,296	3.4 (2.2–4.7)	43,107,418	20.4 (17.8–23.0)	0.17	<0.001
**Injury intent**						
Unintentional or undetermined	413,518	56.4 (51.5–61.3)	193,843,811	91.7 (90.0–93.3)	0.62	<0.001
Assault	253,561	34.6 (29.2–39.9)	13,733,851	6.5 (4.9–8.1)	5.32	<0.001
Self-harm	66,468	9.1 (7.1–11.0)	3,920,256	1.9 (1.6–2.2)	4.89	<0.001
**Mechanism of injury**						
Struck by or against an object	323,085	44.0 (38.7–49.4)	31,057,444	14.7 (13.6–15.7)	3.00	<0.001
Other	149,666	20.4 (17.3–23.5)	86,763,946	41.0 (39.1–42.9)	0.50	<0.001
Fall	145,096	19.8 (17.4–22.2)	62,093,113	29.4 (27.1–31.6)	0.67	<0.001
Overdose or poisoning	56,319	7.7 (5.7–9.7)	14,602,531	6.9 (5.9–7.9)	1.11	0.388
Cut or pierce	48,405	6.6 (5.2–8.0)	14,359,137	6.8 (6.3–7.3)	0.97	0.814
Inhalation or suffocation	6,881	0.9 (0.5–1.3)	400,448	0.2 (0.1–0.2)	4.96	<0.001
Fire or burn	4,096	0.6 (0.4–0.7)	2,221,300	1.1 (1.0–1.1)	0.53	<0.001
**Disposition**						
Treated and released	585,293	79.8 (76.4–83.2)	176,024,926	83.3 (80.0–86.7)	0.96	0.053
Transferred or hospitalized	126,702	17.3 (13.8–20.8)	27,899,796	13.2 (11.4–15.1)	1.31	<0.001
Other (AMA/LWBS, Unk, or observation)	21,552	2.9 (1.3–4.6)	7,308,060	3.5 (0.7–6.2)	0.85	0.393

**Abbreviations:** AMA = against medical advice; LWBS = left without being seen; RP = ratio of proportions; Unk = unknown.

* Numbers are weighted.

^†^ The nonincarcerated sample was used as the referent group for this analysis.

Among incarcerated adults, the mechanism of injury for ED visits differed by age group (Table 2). When compared with ED visits by incarcerated adults aged ≥65 years, a higher proportion of ED visits by those aged 18–24 years resulted from being struck by or against an object or being cut or pierced, and a lower proportion of visits resulted from a fall. A higher proportion of ED visits by incarcerated adults aged 18–24 years was attributable to assault or self-harm compared with those by incarcerated adults aged ≥65 years. A lower proportion of ED visits in this youngest age group resulted in hospitalization or transfer to another hospital.

**TABLE 2:** Characteristics of nonfatal injury-related emergency department visits among incarcerated adults, by age group — National Electronic Injury Surveillance System–All Injury Program, United States, 2010–2019

Characteristic	Age group, yrs											
	18–24		25–34		35–44		45–54		55–64		≥65	
	% (95% CI)	RP (95% CI)	% (95% CI)	RP (95% CI)	% (95% CI)	RP (95% CI)	% (95% CI)	RP (95% CI)	% (95% CI)	RP (95% CI)	% (95% CI)	RP (95% CI)
**Injury intent**												
Unintentional or undetermined	51.6 (46.6–56.6)	0.6* (0.6–0.6)	52.6 (47.3–57.9)	0.6* (0.6–0.7)	54.8 (50.8–58.9)	0.6* (0.6–0.7)	63.7 (57.6–69.8)	0.8* (0.7–0.8)	66.2 (58.6–73.8)	0.8* (0.7–0.8)	86.6 80.0–93.2)	Ref
Assault	39.1 (33.1–45.1)	3.1* (2.2–4.4)	36.3 (29.9–42.6)	2.9* (2.1–4.1)	35.3 (30.9–39.7)	2.8* (2.0–4.0)	30.3 (24.8–35.7)	2.4* (1.7–3.4)	29.0 (21.4–36.7)	2.3* (1.6–3.3)	12.6 (5.8–19.4)	Ref
Self-harm	9.3 (6.8–11.9)	11.5* (3.3–40.3)	11.1 (7.9–14.3)	13.7* (4.0–47.6)	9.9 (7.2–12.5)	12.2* (3.5–42.4)	6.0 (4.7–7.4)	7.5* (2.1–26.4)	4.8 (2.9–6.7)	5.9* (1.6–21.7)	—^†^	Ref
**Mechanism of injury**												
Struck by or against an object	51.8 (45.5–58.1)	2.7* (2.0–3.5)	45.8 (39.7–52.0)	2.4* (1.8–3.5)	43.4 (38.5–48.4)	2.2* (1.7–2.9)	37.7 (32.6–42.8)	1.9* (1.5–2.5)	38.0 (31.3–44.7)	2.0* (1.5–2.6)	19.5 (12.2–26.8)	Ref
Other	18.3 (14.9–21.7)	1.0 (0.8–1.3)	21.0 (17.4–24.7)	1.2 (0.9–1.5)	21.6 (18.3–25.0)	1.2 (0.9–1.6)	21.6 (17.3–25.9)	1.2 (0.9–1.6)	18.0 (12.5–23.4)	1.0 (0.7–1.4)	18.4 (13.4–23.3)	Ref
Fall	14.2 (12.2–16.2)	0.3* (0.2–0.3)	16.3 (13.4–19.3)	0.3* (0.3–0.4)	18.8 (16.5–21.0)	0.4* (0.3–0.4)	23.9 (19.9–27.8)	0.5* (0.4–0.5)	33.5 (27.2–39.7)	0.6* (0.5–0.7)	53.6 (43.5–63.7)	Ref
Overdose or poisoning	7.8 (4.6–11.0)	1.4 (0.8–2.5)	7.3 (5.0–9.6)	1.4 (0.8–2.3)	7.8 (5.6–10.1)	1.5 (0.8–2.5)	9.7 (7.0–12.5)	1.8* (1.0–3.2)	5.9 (3.1–8.7)	1.1 (0.6–2.0)	—	Ref
Cut or pierce	6.7 (5.0–8.4)	3.5* (1.5–8.1)	7.9 (5.7–10.2)	4.2* (1.8–9.5)	6.8 (5.1–8.6)	3.6* (1.6–8.3)	5.1 (3.6–6.7)	2.7* (1.2–6.4)	3.5 (1.8–5.2)	1.9 (0.7–4.7)	—	Ref
Inhalation or suffocation	—	—	—	—	1.1 (0.5–1.7)	3.3 (0.4–24.3)	—	—	—	—	—	Ref
Fire or burn	—	—	0.6 (0.3–1.0)	0.7 (0.2–2.9)	—	—	—	—	—	—	—	Ref
**Disposition**												
Treated and released	86.7 (83.8–89.5)	1.2* (1.1–1.3)	80.3 (76.6–84.0)	1.1* (1.0–1.2)	79.3 (75.0–83.7)	1.1 (1.0–1.2)	74.5 (69.3–79.8)	1.0 (0.9–1.1)	70.3 (65.4–75.2)	0.9 (0.9–1.0)	74.7 (68.1–81.3)	Ref
Transferred or hospitalized	10.5 (8.3–12.7)	0.5* (0.3–0.6)	16.5 (12.8–20.2)	0.7* (0.6–0.9)	18.3 (13.7–22.9)	0.8* (0.6–1.0)	21.6 (16.6–26.6)	0.9 (0.7–1.2)	27.2 (22.3–32.0)	1.2 (0.9–1.5)	23.7 (16.8–30.5)	Ref
Other (AMA/LWBS, Unk, or observation)	**—**	—	—	—	—	—	3.9 (1.8–5.9)	2.4 (1.0–6.0)	—	—	—	Ref

**Abbreviations:** AMA = against medical advice; LWBS = left without being seen; Ref = referent group; RP = ratio of proportions; Unk = unknown.

* Statistically significant at p<0.05.

^†^ Dashes indicate that values were suppressed because of one of the following criteria: small sample size (<20 cases), a weighted estimate <1,200, or a coefficient of variation >30%.

Reasons for ED visits by incarcerated adults also differed by sex (Table 3). Compared with ED visits by incarcerated men, a lower proportion of visits by incarcerated women resulted from being struck by or against an object, and a higher proportion were for fall-related injuries and overdose or poisoning. The proportion of ED visits attributable to assault by incarcerated women (20.0%) was lower than that by incarcerated men (37.4%).

**TABLE 3:** Characteristics of nonfatal injury-related emergency department visits among incarcerated adults, by sex — National Electronic Injury Surveillance System–All Injury Program, United States, 2010–2019

Characteristic	Men		Women	
	% (95% CI)	RP	% (95% CI)	RP
**Injury intent**				
Unintentional or undetermined	53.5 (48.8–58.1)	Ref	71.3 (68.0–74.7)	1.3* (1.3–1.4)
Assault	37.4 (32.0–42.8)	Ref	20.0 (17.4–22.6)	0.5* (0.5–0.6)
Self–harm	9.1 (6.8–11.4)	Ref	8.7 (6.4–11.0)	1.0 (0.8–1.2)
**Mechanism of injury**				
Struck by or against an object	46.6 (40.9–52.2)	Ref	31.1 (27.8–34.5)	0.7* (0.6–0.7)
Other	19.1 (16.0–22.1)	Ref	27.2 (23.8–30.6)	1.4* (1.3–1.6)
Fall	19.0 (16.7–21.3)	Ref	23.8 (18.9–28.7)	1.3* (1.1–1.4)
Overdose or poisoning	7.1 (4.9–9.3)	Ref	10.7 (8.0–13.5)	1.5* (1.3–1.8)
Cut or pierce	6.9 (5.2–8.6)	Ref	5.2 (3.7–6.8)	0.8* (0.6–1.0)
Inhalation or suffocation	0.9 (0.5–1.4)	Ref	—^†^	—
Fire or burn	0.5 (0.4–0.6)	Ref	—	—
**Disposition**				
Treated or released	79.5 (75.8–83.2)	Ref	81.4 (78.1–84.6)	1.0 (1.0–1.1)
Transferred or hospitalized	17.8 (13.9–21.6)	Ref	14.8 (11.9–17.7)	0.8* (0.7–1.0)
Other (AMA/LWBS, Unk, or observation)	2.8 (1.2–4.4)	Ref	3.8 (1.8–5.8)	1.4* (1.0–1.9)

**Abbreviations:** AMA = against medical advice; LWBS = left without being seen; Ref = referent group; RP = ratio of proportions; Unk = unknown.

* Statistically significant at p<0.05.

^†^ Dashes indicate that values were suppressed because of either small sample size (<20 cases) or a weighted estimate <1,200.

## Discussion

During the study period, an estimated 750,000 ED visits by incarcerated adults and >200 million by nonincarcerated adults occurred. The proportion of ED visits for assault and self-harm was five times as high among incarcerated adults than among nonincarcerated adults. A higher proportion of ED visits by incarcerated adults resulted from being struck by or against an object, compared with ED visits by nonincarcerated adults. Among incarcerated adults with injury-related ED visits, there were differences in injury mechanisms by age group and by sex. This study is the first to present national estimates of nonfatal injury-related ED visits by incarcerated adults in the United States. 

A majority of ED visits among incarcerated adults were made by men and persons aged ≤45 years, likely reflecting the makeup of the incarcerated population (*1*,*2*). However, because older adults are the fastest growing segment of prison populations (*6*,*7*), it is notable that a higher proportion of ED visits for unintentional injuries, including falls, and a higher proportion of ED visits resulting in hospitalization occurred among incarcerated adults aged ≥65 years than occurred among younger incarcerated adults. A higher proportion of ED visits among incarcerated women were related to poisoning and falls, and a lower proportion were related to assault than were those by incarcerated men. These findings illustrate the importance of age- and sex-specific injury prevention strategies for incarcerated adults.

Approximately 50,000 assaults occurring within public correctional facilities are reported annually (*7*). Persons aged ≥18 years detained in jails were twice as likely to die by suicide in 2019 compared with persons in the overall U.S. adult population (*8*). The higher proportion of assault- and self-harm–related ED visits among incarcerated persons, particularly among younger adults and men, points to the need for the development and implementation of violence and suicide prevention strategies that consider the intersectional factors related to incarceration.

The findings in this report are subject to at least five limitations. First, there are differences in health care access for incarcerated persons, which might partially explain the observed differences. For example, some correctional facilities have the capacity to provide health care on-site, and therefore, a subset of injuries among incarcerated adults might not be represented in these findings. The decision to seek ED care is made by authorities rather than patients, which could affect the types of ED visits made by incarcerated persons and be biased toward more serious injuries. Second, it is unclear how the proximity of NEISS-AIP–participating hospitals to correctional facilities affects national estimates presented in this study. Third, classification of incarcerated status was based on text narratives written by NEISS-AIP data abstractors, which might have led to misclassification of incarceration status. Fourth, it was not possible to determine when the injury occurred for incarcerated cases; therefore, data include injuries that could have occurred before incarceration. Finally, data on race and ethnicity were not presented because these data in NEISS-AIP are incomplete; however, U.S. Department of Justice statistics consistently demonstrate that Black or African American men are disproportionately overrepresented in the correctional system *(**1**,**2**)* stemming from upstream factors, particularly structural racism (*9*), and are therefore likely overrepresented in these data.

Nearly one in every 100 persons in the United States is in a prison or jail (*10*), and this study found that characteristics of ED visits by incarcerated adults differ from those by nonincarcerated adults. These differences suggest that setting-appropriate risk-prevention strategies that account for the conditions experienced while incarcerated could help prevent injuries among incarcerated persons. Increased availability of community- and facility-level resources for comprehensive mental health services and creating protective environments could help mitigate the risk for self-harm and violence associated with incarceration. CDC has created technical packages and resources that outline evidence-based strategies for communities for preventing suicide,^¶¶^ interpersonal and community violence,*** overdose,^†††^ and falls^§§§^; tailoring these strategies and developing interventions for the jail and prison setting with age-appropriate and sex-specific recommendations might reduce injuries and ED visits in this population.

**Summary**

**What is already known about this topic?**

Incarcerated adults experience disproportionate negative health outcomes compared with the general adult population, including unintentional and violence-related injuries.

**What is added by this report?**

The proportion of nonfatal injury-related emergency department (ED) visits by incarcerated adults resulting from assault or self-harm was five times as high as those among nonincarcerated adults. Among incarcerated adults, men and persons aged <65 years had the highest proportions of assault-related ED visits. Falls accounted for the most ED visits among incarcerated adults aged ≥65 years. A higher proportion of ED visits by incarcerated women than incarcerated men was for overdose or poisoning.

**What are the implications for public health practice?**

Tailoring injury prevention efforts for incarcerated adults with age- and sex-specific strategies might reduce injuries and ED visits in this population.

## Footnotes

* Assault was defined as assault or legal intervention.

†
https://www.cpsc.gov/s3fs-public/2001d010-6b6.pdf

§ ED visits among persons brought in by law enforcement before being booked in a jail or prison (e.g., immediately after arrest) or among persons who sustained an injury during detention-associated transportation were excluded from the case definition. Only persons brought directly to the ED from either a prison or a jail were included in this analysis. Injuries could have been sustained before incarceration. Occupational injuries were removed to exclude staff members injured while working in jails or prisons and were removed from nonincarcerated cases for consistency.

¶ “Jail,” “prison,” “corrections,” “correctional,” “incarcerate,” “incarcerated,” “incarceration,” “convict,” “inmate,” “detention,” “detain,” “detainee,” “detained,” and “police cell,” were used for inclusion, and other relevant search terms such as “custody,” “custody dispute,” “cell phone,” and “cellulitis” were used to help identify cases for exclusion.

** Injuries were categorized by intent (unintentional/undetermined, assault/legal intervention, or self-harm). Injuries were also categorized by mechanism of injury (fall, cut/pierce, struck by/against an object, inhalation/suffocation, overdose/poisoning, fire/burn, or other). Other includes injuries related to motor vehicles (occupant, motorcyclist, pedal cyclist, pedestrian, and other transport, which includes traffic-related, non–traffic-related, and unknown transportation injuries), overexertion, drowning/submersion, machinery, foreign body, dog bite, other bite/sting, firearm gunshot, bb/pellet gunshot, natural/environmental, other specified, and unknown/unspecified.

†† Disposition was categorized as treated and released, transferred to another hospital or hospitalized, or other. Other includes patients who left without being seen, left against medical advice, were held for observation, or had unknown disposition.

§§ 45 C.F.R. part 46.102(l)(2), 21 C.F.R. part 56; 42 U.S.C. Sect. 241(d); 5 U.S.C. Sect. 552a; 44 U.S.C. Sect. 3501 et seq.

¶¶ https://www.cdc.gov/violenceprevention/communicationresources/pub/technical-packages.html; https://www.cdc.gov/suicide/pdf/suicideTechnicalPackage.pdf

***
https://www.cdc.gov/violenceprevention/communityviolence/index.html ; https://www.cdc.gov/violenceprevention/communityviolence/prevention.html

†††
https://www.cdc.gov/drugoverdose/strategies/index.html

§§§
https://www.cdc.gov/falls/programs/community_prevention.html
